# The Yield of Routine Post-Operative Doppler Ultrasound to Detect Early Post-Liver Transplantation Vascular Complications

**DOI:** 10.3389/ti.2023.11611

**Published:** 2023-11-29

**Authors:** Iulia Minciuna, Caroline den Hoed, Adriaan J. van der Meer, Milan J. Sonneveld, Dave Sprengers, Robert J. de Knegt, Jeroen de Jonge, Raoel Maan, Wojciech G. Polak, Sarwa Darwish Murad

**Affiliations:** ^1^ Department of Gastroenterology and Hepatology, Erasmus MC Transplant Institute, Erasmus MC University Medical Center Rotterdam, Rotterdam, Netherlands; ^2^ Department V- Gastroenterology, University of Medicine and Pharmacy “Iuliu Hatieganu”, Cluj-Napoca, Romania; ^3^ Regional Institute of Gastroenterology and Hepatology “O. Fodor”, Cluj-Napoca, Romania; ^4^ Department of Surgery, Division of HPB and Transplant Surgery, Erasmus MC Transplant Institute University Medical Center Rotterdam, Rotterdam, Netherlands

**Keywords:** routine Doppler ultrasound, hepatic artery thrombosis, portal vein thrombosis, outflow obstruction, liver transplantation

## Abstract

Early detection of liver transplantation (LT) vascular complications enables timely management. Our aim was to assess if routine Doppler ultrasound (rDUS) improves the detection of hepatic artery thrombosis (HAT), portal vein thrombosis (PVT) and hepatic venous outflow obstruction (HVOO). We retrospectively analysed timing and outcomes, number needed to diagnose one complication (NND) and positive predictive value (PPV) of rDUS on post-operative day (POD) 0,1 and 7 in 708 adult patients who underwent primary LT between 2010–2022. We showed that HAT developed in 7.1%, PVT in 8.2% and HVOO in 3.1% of patients. Most early complications were diagnosed on POD 0 (26.9%), 1 (17.3%) and 5 (17.3%). rDUS correctly detected 21 out of 26 vascular events during the protocol days. PPV of rDUS was 53.8%, detection rate 1.1% and NND was 90.5. Median time to diagnosis was 4 days for HAT and 47 days for PVT and 21 days for HVOO. After intervention, liver grafts were preserved in 57.1%. In conclusion, rDUS protocol helps to detect first week’s vascular events, but with low PPV and a high number of ultrasounds needed.

## Graphical Abstract

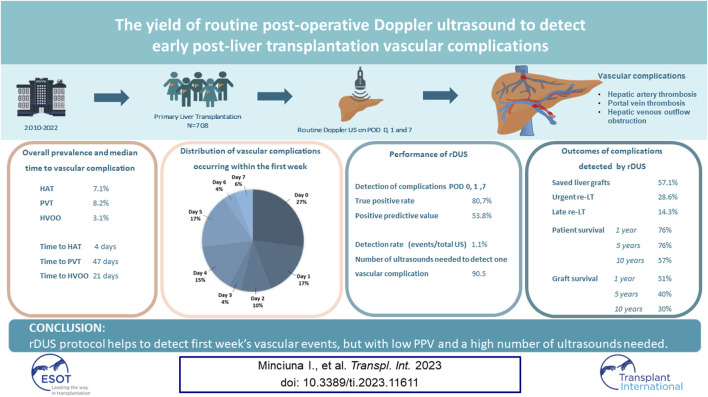

## Introduction

With an impressive evolution over the past 50 years, liver transplantation (LT) has changed the quality of life and survival of many patients with acute liver failure, end-stage liver disease and hepatocellular carcinoma. The continuous improvements in patient selection, surgical techniques, perioperative management and immunosuppression have led to an increased liver graft and patient survival over time. Although the increased experience and proficiency has resulted in a change of the incidence, nature and outcome of vascular complications, they remain the most feared complications of LT as they can lead to graft dysfunction or even graft loss and patient death.

Hepatic artery thrombosis (HAT) is an infrequent (incidence around 5%) but potentially devastating [1] complication with high morbidity and likelihood of graft failure or even mortality in the early post-LT setting. Moreover, as the bile ducts are particularly susceptible to hypoxia, HAT often leads to ischemic biliary complications, with subsequent secondary infection, abscesses and necrosis [2]. With an incidence of almost 3%, portal vein thrombosis (PVT) can cause graft failure, graft ischaemia, intestinal ischaemia, and persistence or recurrence of portal hypertension with ascites and variceal bleeding [3]. Hepatic venous outflow obstruction (HVOO), due to thrombosis or stenosis at the level of the hepatic veins or cavo-cavostomy, is an infrequent complication (incidence less than 3%) [3] that can lead to graft dysfunction and graft failure, with mortality rates reaching up to 24% [4, 5].

Early detection of these vascular complications is of paramount importance for timely management in order to achieve favourable outcomes in LT patients. Hereto, there are well-accepted standards including early post-LT sequential ultrasound evaluation of the vascular patency. The specifics regarding implementing them, however, differ according to centre as there are ongoing debates regarding the frequency and use of routine liver vessel assessment.

The aim of this study was to assess if routine Doppler ultrasound (rDUS), performed at day 0, 1 and 7 at our institution, improves the detection rate of early post LT vascular complications (i.e., HAT, PVT and HVOO).

## Patients and Methods

### Study Population

All adult patients who underwent LT between January 2010 and September 2022 at our transplant centre were included. A routine Doppler ultrasound protocol was implemented in 2010 and consists of performing abdominal Doppler ultrasound by hepatologists with extensive ultrasonography experience during day 0 (usually within 1 h post-surgery), day 1 and day 7 post LT. We used the Hitachi Hi Version Preirus^®^ from 2010–2018 and the Philips Epiq 7G^®^ from 2018–2022. Ultrasound examination included the evaluation of the hepatic artery by colour Doppler to assess patency and by pulse-wave technology to evaluate the wave pattern and calculate the resistive index (RI) of the hepatic artery at the level of the hilum and the right and left branch. The arterial anastomosis was considered patent when there was a normal wave pattern (i.e., no parvus tardus) and the RI was greater than 0.5 at all locations. The portal vein patency and flow direction (hepatofugal/hepatopetal) were evaluated by colour Doppler and the flow velocity by pulse-wave technology. The portal vein anastomosis was considered patent when the flow direction was hepatopetal, there was no intraluminal material and the acceleration between the pre-anastomotic and post-anastomotic portal vein velocity was less than three-fold. The patency and phase of three hepatic veins and the cavo-cavostomy were evaluated by colour Doppler. The outflow was considered sufficient when there was flow in all three hepatic veins and the cavo-cavostomy, the wave pattern in the hepatic veins were either triphasic or biphasic and the diameter of the hepatic veins was <10 mm. Whenever the patency of the hepatic artery, portal vein or hepatic vein was not considered sufficient or was debated, patients underwent a confirmatory CT (Computed Tomography). After discharge, all patients underwent life-long complete follow-up at our centre. All participants signed an informed consent before LT for retrospective data collection. The study adhered to the Declaration of Helsinki and is in concordance with the principles of the Declaration of Istanbul on Organ Trafficking and Transplant Tourism.

### Data Collection and Definitions

The following recipient variables were collected at time of transplantation: age, gender, BMI, indication for LT, calculated Model for End-Stage Liver Disease (MELD) score at time of LT, type of graft [i.e., donation after brain death (DBD), donation after cardiac death (DCD) or living donor liver transplantation (LDLT)]. We collected data on the occurrence of HAT, PVT and HVOO during the first 7 days post-LT and at any time thereafter. HAT was defined as a thrombotic occlusion of hepatic artery that led to the absence of hepatic artery signal at the hilum or the intrahepatic arterial branches on Doppler Ultrasound and/or a non-enhancing filling defect on contrast-enhanced CT scan. PVT was defined as thrombotic occlusion of the portal vein that led to a filling defect of portal blood flow on Doppler Ultrasound and/or a portal non-enhancing filling defect on contrast enhanced CT. HVOO included either thrombosis or stenosis at the level of caval anastomosis or hepatic veins diagnosed by US, CT or venography. For each vascular complication, we collected data on the first radiological imaging technique used to diagnose the complication (i.e., DUS or CT) as well as the main indication for performing the imaging. These indications were either routine imaging on postoperative day 0, 1 or 7 or a clinical indication, defined as clinical deterioration, worsening graft function, ascites or other signs of portal hypertension, unexplained fever or abdominal pain, or hemodynamic changes. All patients with a rDUS suggestive of a vascular complication underwent subsequent CT to confirm the diagnosis. Furthermore, data on type of therapeutic intervention (i.e., surgical revascularization, endovascular radiological treatment, anticoagulation/antiplatelet treatment only and conservative treatment), total duration of hospitalization, need for re-LT within 7 days from liver vascular complication (i.e., urgent re-LT), and re-LT at any time point (i.e., re-LT) were obtained.

### Statistical Analysis

Statistical analyses were descriptive. Quantitative variables were expressed as medians with extreme values (range) and compared using Student’s t-test or Wilcoxon test as appropriate. Qualitative variables were expressed as numbers and percentages and compared using Chi-square or Fisher’s exact tests, as appropriate.

Primary outcomes were the frequency of HAT, PVT and HVOO at different time points within the first week. Secondary outcomes were the use of surgical or interventional therapies as well as graft and patient survival.

The diagnostic performance of protocol routine Doppler ultrasound in detecting HAT, PVT and outflow obstruction was expressed in terms of positive predictive value with CT as gold standard. Detection rate of rDUS was defined as number of vascular events detected/total number of ultrasounds performed. The number needed to image in order to detect one complication was calculated as (1/detection rate of rDUS). Due to the fact that a negative rDUS was not routinely followed by CT, it was not possible to calculate sensitivity, specificity and negative predictive value. All statistical analyses were performed using commercially available statistical software (SPSS Inc., Chicago, IL). A *p*-value of <0.05 was considered statistically significant.

## Results

### Patient and Liver Transplantation Characteristics

In total, 708 patients underwent primary liver transplantation and were followed for a median of 3.69 years (range 0–12.4). The baseline characteristics of the study population are presented in Table 1.

**TABLE 1:** Baseline characteristics of 708 patients undergoing primary liver transplantation at our institution between 2010–2022.

Variables	Total population *n* = 708
Recipient sex (male)	445 (62.9%)
Recipient age at LT (years)	55 (17, 72)
BMI (kg/m^2^)	25.5 (15.36–46.78)
Liver disease aetiology	
Viral	117 (16.5%)
Alcohol related liver disease	106 (15%)
Steatotic liver disease	70 (9.9%)
Biliary	178 (25.1%)
Autoimmune	20 (2.8%)
Acute liver failure	52 (7.3%)
Inherited metabolic liver diseases	36 (5.1%)
Vascular liver diseases	7 (1%)
Cryptogenic	28 (4%)
Other	94 (13.3%)
Hepatocellular carcinoma	239 (33.8%)
MELD Score at LT	22 (6–40)
Pre-LT PVT	73 (10.3%)
Type of graft	
DBD	419 (59.2%)
DCD	256 (36.2%)
Living Donor	31 (4.4%)
Domino	2 (0.3%)
Use of machine perfusion	108 (15.3%)
Surgical characteristics	
PVT at time of implantation	67 (9.5%)
Performance of portal thrombectomy	57 (8.1%)
Use of portal conduits	11 (1.6%)
Portal vein reconstruction	21 (3%)
End-to-end portal anastomosis	678 (98.7%)
Hepatic artery reconstruction	118 (16.7%)
Arterial re-do	35 (4.9%)
Intraoperative hepatic artery thrombosis	23 (3.2%)
Piggy-back caval anastomosis	681 (99.3%)

LT, liver transplantation; ALD, alcoholic liver disease; MEL, model for end-stage liver disease; DBD, donation after brain death; DCD, donation after cardiac death; rDUS, routine Doppler ultrasound.

Most of the patients were male (*n* = 445; 62.9%), with a median age of 55 (17–72) years, BMI of 25.5 kg/m^2^ (15.3–46.7) and laboratory MELD score of 22 (6,40) at the time of first LT. Main LT indications were HCC (*n* = 239; 33.8%) and cholestatic liver disease (*n* = 178; 25.1%), followed by viral hepatitis in 117 (16.5%) and alcoholic liver disease in 106 (15%) patients. Machine perfusion was performed in 15.3%. Overall, 1, 5 and 10 years patient survival was 92.1% (95% CI: 91.1–93.1), 80.8% (95% CI: 79.1–82.5), and 68.2% (95% CI: 65.2–71.2). Graft survival was 87.5% (95% CI: 86.2–88.8), 73.9% (95% CI: 72–75.8), and 62.8% (95% CI: 60–65.6), respectively.

### Vascular Complications Following Liver Transplantation

**FIGURE 1:**
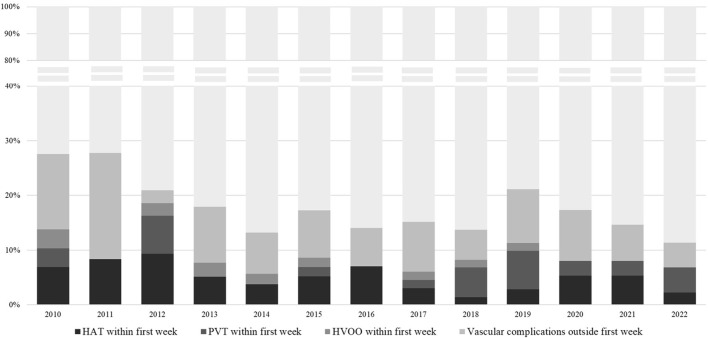
Percentage of vascular complications at any time, HAT within the first week, PVT within the first week and HVOO within the first week, per calendar year of transplantation. rDUS, routine Doppler ultrasound; HAT, hepatic artery thrombosis; PVT, portal vein thrombosis; HVOO, hepatic venous outflow obstruction.

Of the entire population, 112 (15.8%) patients developed at least one vascular complication within a median of 0.33 months (range 0–95.4) after LT, of whom 52 (46.4%) within the first week. The rate of vascular complications during the study time period is shown in Figure 1. The rate of any vascular complication was 18% in 2010–2016, and 14.7% in 2017–2022 (*p* = 0.28). Median time to diagnosis of any vascular event was 10 days (0–2,864), for HAT was 4 days (0–1,382), for PVT 47 days (0–2,864) and HVOO 21 days (0–1933). In total, 50 (7.1%) patients developed HAT, of whom 34 (68%) within the first week. PVT was found in 58 patients (8.2%), 21 of whom during the first week (36.2%), and HVOO was identified in 22 patients (3.1%) (i.e., *n* = 4 caval stenosis, *n* = 16 thrombosis in hepatic vein(s), *n* = 2 thrombosis in the IVC), 8 of those (36.3%) within the first week.

The indication for the diagnostic CT within the first 7 days was a suspected vascular complication by rDUS in 40.4% of cases and laboratory changes in 34.6% of cases. Outside of the first week, imaging was either driven by clinical symptoms, laboratory changes or incidental, i.e., during re-evaluation for other indications (e.g., follow-up CT for HCC recurrence) (see Table 2).

**TABLE 2:** Vascular complications following liver transplantation in the overall population.

Details on vascular complications	Total population *n* = 708
Vascular complications at any time point	112 (15.8%)
Vascular complications on Day 0, 1, 7	26 (3.7%)
Vascular complications within the first week	52 (7.3%)
Indication for diagnostic CT within first week	*n* = 52
rDUS	21 (40.4%)
Laboratory changes	18 (34.6%)
Abdominal complaints	10 (19.2%)
Fever/infection	3 (5.8%)
Indication for diagnostic CT outside first week	*n* = 60
Laboratory changes	15 (2%)
Abdominal complaints	17 (28.3%)
Fever/infection	14 (23.3%)
Incidental on imaging for other reasons	14 (23.3%)
Duration of hospitalization for patients with complications within the first week (days)	25 (10, 186)
Time to HAT diagnosis (days)	4 (0, 1,382)
Time to PVT diagnosis (days)	47 (0, 2,864)
Time to HVOO diagnosis (days)	21 (0, 1,933)
Vascular complications within 7 days to 3 months	30 (4.2%)
Vascular complications within 3 months to 1 year	10 (1.4%)
Vascular complications outside first year	20 (2.8%)

rDUS, routine Doppler ultrasound; iCT, imaging upon indication; HAT, hepatic artery thrombosis; PVT, portal vein thrombosis; HVOO, hepatic venous outflow obstruction; CT, computer tomography.

**FIGURE 2:**
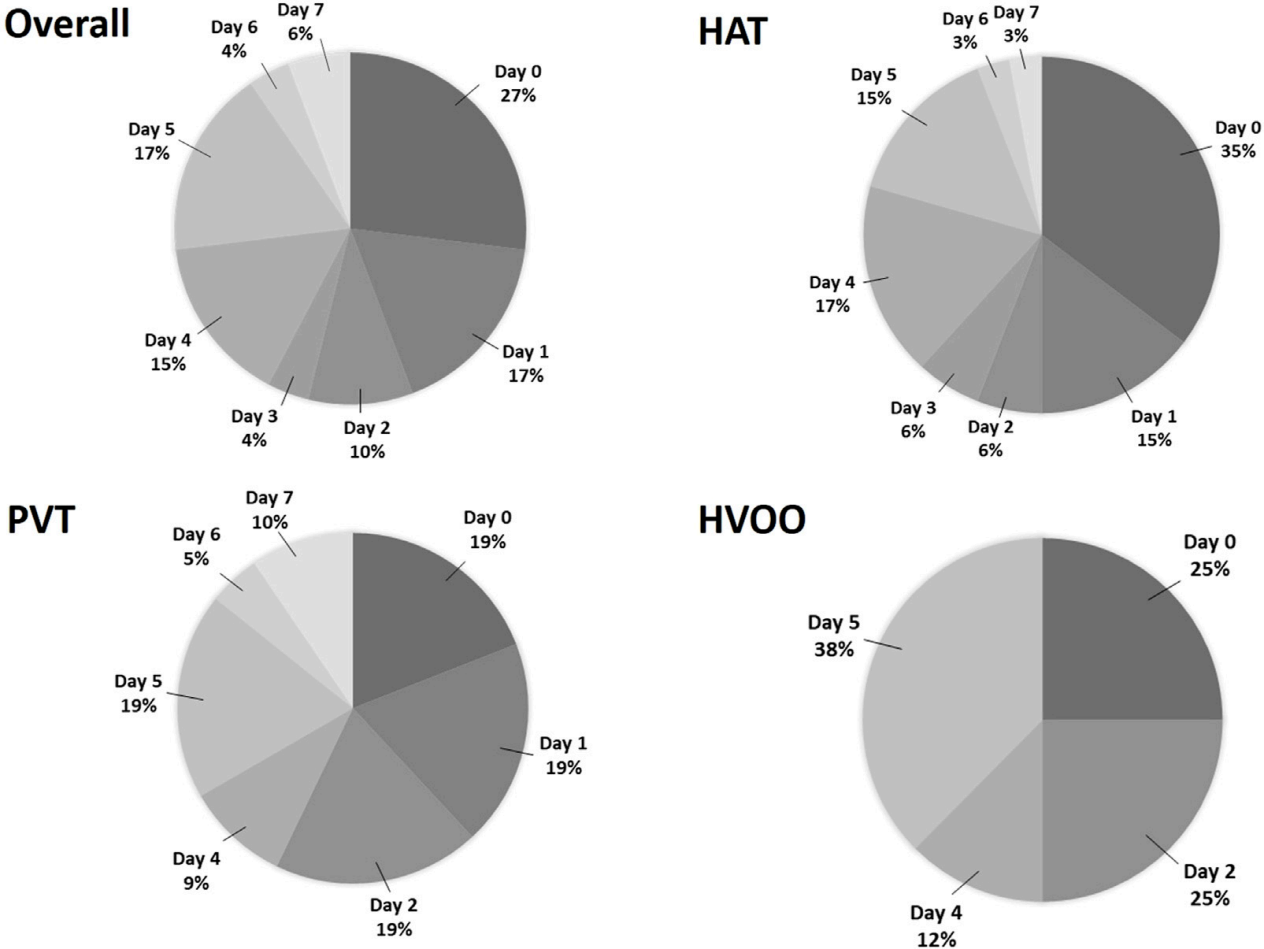
Pie chart showing all (*n* = 52) complications, all HAT (*n* = 34), all PVT (*n* = 21) and all HVOO (*n* = 8), occurring within the first week separated by postoperative day. HAT, hepatic artery thrombosis; PVT, portal vein thrombosis; HVOO, hepatic venous outflow obstruction.

In the first week, 34 patients were diagnosed with HAT (68% of all vascular events), 21 with PVT (36.2%) and 8 with HVOO (36.3%). Eleven of these (21.1%) had more than 1 vascular complication at the same time. Most of the vascular complications during the first week were diagnosed on post-operative day (POD) 0 (*n* = 14; 26.9%), 1 (*n* = 9; 17.3%) and 5 (*n* = 9; 17.3%), followed by POD 4 (*n* = 8; 15.4%), while at POD 7, only 3 patients were diagnosed (5.8%) (see Figure 2).

### Performance of the rDUS Protocol

In total, 26 patients (3.7%) had a vascular event on day 0, 1 and 7, which included 18 (69.2%) HAT, 10 (38.5%) PVT and 2 (7.7%) patients with HVOO. Twenty-one vascular events (80.8%) were detected by rDUS, and the remaining five were initially missed but detected on CT by indication the same day (*n* = 3 for laboratory changes and *n* = 2 for abdominal complaints). Therefore, the diagnostic yield of rDUS was 80.7% [i.e., 21 (positive on rDUS and confirmatory CT)/26 (total positive on CT)]. In 18 patients, a vascular complication was suspected on rDUS, but not confirmed on the following CT (i.e., false positive), hence the positive predictive value was 53.8% (21/39). The total number of routine Doppler ultrasounds performed in the 708 patients was 1900 (i.e., median 3 per patient; range 1–3). Therefore, the vascular complication detection rate of rDUS was 21/1900 (1.1%) and the minimal number of DUS needed to detect one vascular complication was 90.5 (1900/21).

### Treatment and Outcomes of Vascular Complications Detected by rDUS

**FIGURE 3:**
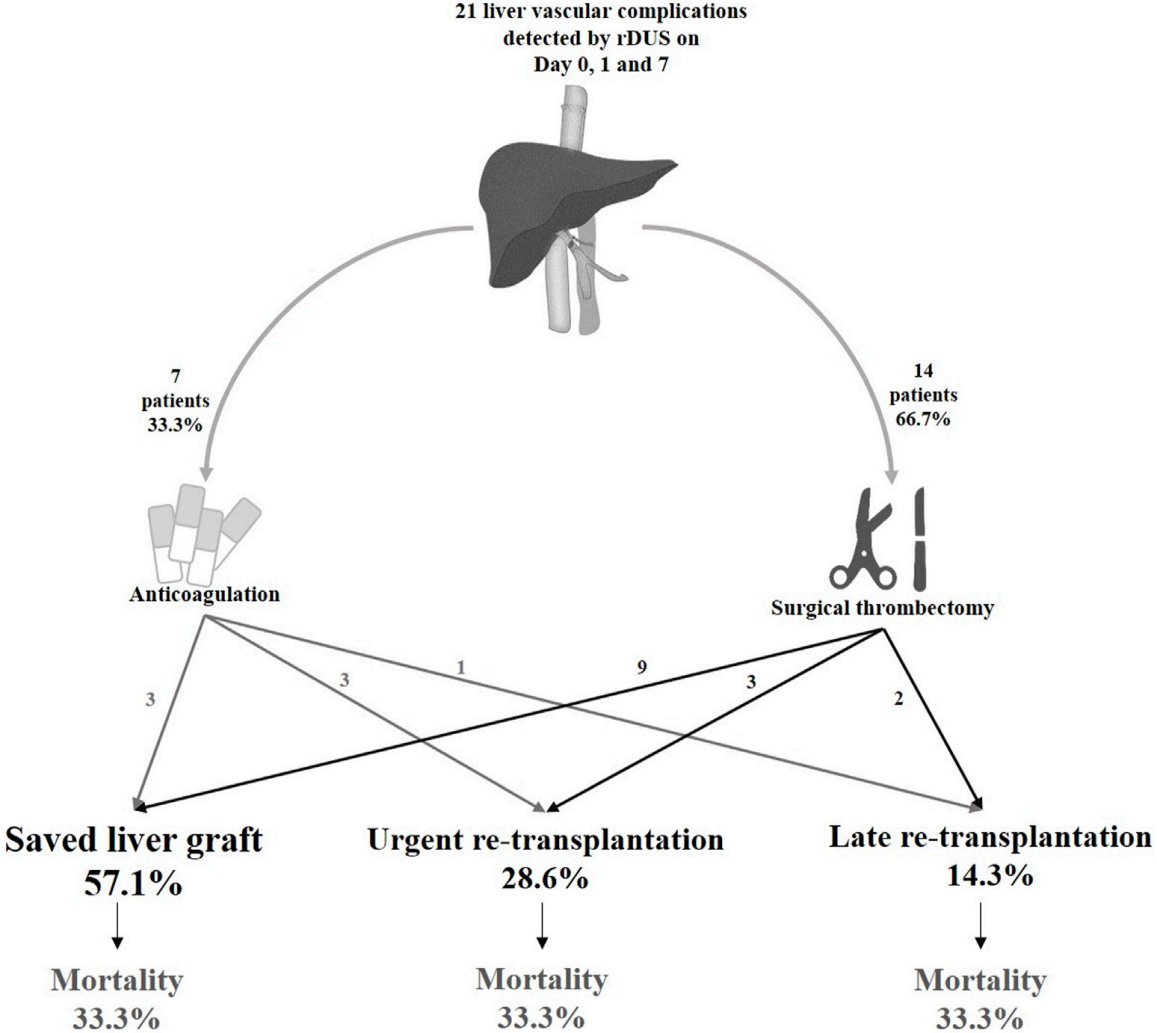
Clinical outcomes of vascular complications diagnosed on days 0, 1 and 7 in the rDUS group. rDUS, routine Doppler ultrasound; iCT, imaging upon indication.

The diagnosis of vascular events during rDUS protocol days led to surgical re-intervention (i.e., thrombectomy and a redo of the anastomosis) in 14 patients (66.7%), while 7 (33.3%) were treated by anticoagulant therapy alone (Figure 3). This approach preserved the graft in 57.1% patients while 28.6% proceeded to urgent re-LT and 14.3% underwent late re-LT.

Among the 14 patients who underwent surgical intervention, nine (64.3%) had a successful outcome (i.e., preserved graft function), and five (35.7%) underwent re-transplantation (3 HAT, 1 PVT and 1 HAT + PVT). Of these, three patients needed urgent re-LT due to unsuccessful portal thrombectomy (*n* = 1), or recurrent occlusion of the hepatic artery (*n* = 2), while two patients required re-LT later due to ischemic biliopathy despite achieving initial arterial recanalization. Among the 7 patients who received anticoagulation alone (*n* = 4 HAT, *n* = 1 PVT, *n* = 1 HAT + PVT and *n* = 1 HAT + HVOO), three (42.9%) patients with HAT had restored graft function, and other four (57.1%) underwent re-LT. Therefore, the majority of all re-transplantations for vascular complications were due to HAT (*n* = 5, 55.5%), followed by PVT (*n* = 2, 22.2%), HVOO + HAT (*n* = 1, 11.1%) and HAT + PVT (*n* = 1, 11.1%).

**FIGURE 4:**
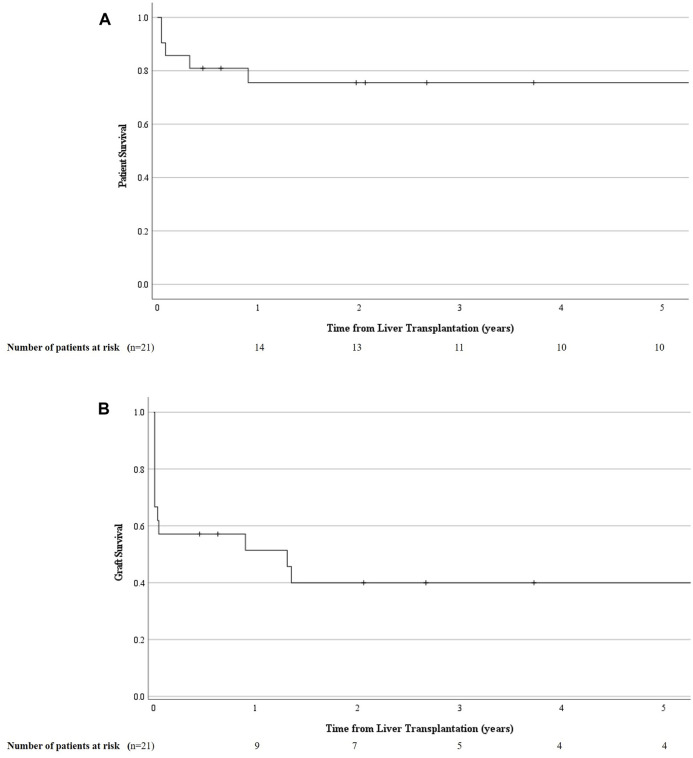
Cumulative patient **(A)** and graft survival **(B)** in those who underwent a primary liver transplantation and developed a liver vascular complication detected by routine Doppler ultrasound (rDUS). rDUS, routine Doppler ultrasound; iCT, imaging upon indication; POD, postoperative day.

In total, 7 (7/21; 33%) patients died, of whom only 1 because of early graft failure within 2 weeks post LT. All others died within a median time of 7.32 months (0.96–108.8) for causes unrelated to the vascular event, i.e., acute on chronic renal failure (*n* = 1), uncontrolled sepsis after second LT (*n* = 2), severe acute pancreatitis (*n* = 1) and malignancy (*n* = 2). Thus, in this group of vascular complications detected by rDUS, the 1, 5 and 10 years patient survival was 76% (95% CI 66.1–85.1), 76% (95% CI 66.1–85.1) and 57% (95% CI: 42.8–70.6); and graft survival was 51% (95% CI: 40.4–62.4), 40% (95% CI: 29–51) and 30% (95 %CI: 17.9–42.1), respectively (Figure 4).

## Discussion

The current study shows that rDUS performed on POD 0, 1 and 7 has a detection rate of 80.7% for vascular complications occurring on these days. However, rDUS is associated with a relatively high number of false positive results (PPV 53.8%) and likewise, a high number of ultrasounds needed to detect one complication (90.5). Given the fact that the vast majority of cases occurred within the first 5 days, our rDUS protocol can be further optimised to include POD 0, 1 and 5 instead.

Early detection of post LT vascular complications has always been considered important as these early complications can lead to fulminant graft failure [6]. Re-transplantation, which usually follows, is an undesired event which puts a further strain on the global shortage of organs and is associated with increased recipient morbidity and mortality [7]. Despite CT angiography being the gold standard for diagnosing HAT and PVT, Doppler ultrasonography is still the most widely used screening imaging technique for assessing the development of hepatic vascular complications in the direct post-LT period because of its non-invasive nature, lack of contrast exposure, accessibility and affordability. In the postoperative period, DUS screening has an important role of detecting liver vascular complications that are still clinically asymptomatic but at the same time may also provide useful information about other, non-vascular complications such as biliary strictures, leakage and fluid collections [8, 9].

Among studies, the highest incidence of early HAT varies from day 1 to day 2 post LT [10, 11], with a median time to detection ranging from 2.5 to 4.9 days [11]. Therefore, increased vigilance is needed during the first week post-LT. Although postoperative DUS is part of the standard screening protocol for vascular complications in many transplant centres, both intraoperatively and postoperatively, the specifics vary considerably [12, 13]. The reported frequency and interval of rDUS range from close monitoring—twice daily for the first week and once daily for the next 7 days [11], to every 3 days for 2 weeks [10], to once on day 1 and once on day 5 [14]—to even continuous monitoring using an implantable DUS for 10 days, with six check-ups per day [8]. In our study population, we showed that most of vascular complications occur during the first week and we have identified the highest number of early post-LT vascular complications at day 0 and 1, followed by day 4 and 5 (totalling up to 90% of the first week complications). Based on the hypothesis that earlier intervention for a vascular complication may benefit graft survival and patient outcome, we considered that rDUS would be better applied at day 0, 1 and 5 instead of 7. This protocol change has now been implemented in our centre based on the current study.

To put some perspective to our findings, we applied the concept of number needed to treat (NNT) to the diagnostic setting. To identify one vascular complication, we needed 91 DUS examinations. Also, the positive predictive value of the DUS was rather disappointing, as almost half of the cases were found to be false positive at the confirmatory CT. Two other studies identified a much higher PPV of 92.3% and 88.9% for rDUS performed daily for either the first 7 days [12] or for the first 2 weeks, respectively [15]. However, it is important to note that we had a low threshold for performing a confirmatory CT. Indeed, we performed a CT not only to confirm an US diagnosis of a clear occlusion or thrombosis but also in situations in which there was any doubt, i.e., when not all vessel patency criteria were met (defined in Methods). This practice may explain our lower positive predictive value. Also, 2 out of 3 days from our rDUS protocol are immediately post-operative and this timeframe is characterized by increased hemodynamic changes due to low cardiac output, arterial spasm and parenchymal oedema [15]. This may hamper DUS visualization of the arterial flow, especially considering the increased portal flow early after LT. As we did not do routine CT in those labelled as negative on DUS, we could not calculate the negative predictive value nor give estimates of sensitivity or specificity. Performing a cost-effectiveness analysis was beyond the scope of our study, however, given the profound impact of re-LT on the patient, on healthcare costs and on the donor organ capacity in general, one could argue that the cost of a relatively cheap DUS examination, despite the high numbers thereof, would still be worthwhile. Ideally, in order to have a definite answer to the diagnostic yield of rDUS compared to performing imaging upon clinical indication only, a randomized trial is needed. Given the now widespread implementation of routine imaging in most transplant centres, and the possibility of missing an important diagnosis, such a trial would be a complicated, and perhaps even unethical, undertaking.

Across time, we identified variations, albeit not significant, in the incidence of vascular complications during the first week. There may be several explanations for this variation. For one, this may be a reflection of variations in the use of more extended criteria grafts including DCD grafts (indeed our centre uses 36.2% DCD’s) or increasing acceptance of candidates with pre-LT PVT or other vascular complexities, which may change the risk of early vascular complications. However, it may also be that the implementation of an rDUS protocol contributed to an increased number of detected complications, like we observed in the early years. Most of these patients were clinically asymptomatic at the time of DUS. It may therefore well be that if we went by clinical judgment alone, these may not have been diagnosed at that time, but perhaps (much) later. This is especially true for days 0 and 1, during which time it is very challenging to know when early signs such as abdominal discomfort or laboratory changes are worrisome and when they are just part of the normal postoperative course. Despite the risk of false positive results during those early days, it seems reasonable to suggest that some form of diagnostic monitoring would still be desirable.

One of the main strengths of our study was that we had a homogeneous single centre cohort in which patients were treated according to predefined protocols and no one was lost to follow-up. Another strength was that we not only examined the diagnostic ability of DUS to detect vascular complications (HAT, PVT and HVOO) but also included data on the clinical consequences of their detection. One of the main limitations of our study is, however, that the true performance of the rDUS protocol could not be determined. This is due to the retrospective nature of the study and the fact that DUS is a standalone imaging modality not routinely requiring the confirmation in case of a normal result. Therefore, we could not calculate the true negative and false negative results.

In conclusion, the majority of post-LT vascular complications occur during the first week. rDUS detects the majority of events during protocol days, but with low PPV and a high number of ultrasounds needed. However, a true clinical benefit in terms of graft and patient’s survival has yet to be shown.

## Data Availability

The raw data supporting the conclusion of this article will be made available by the authors, without undue reservation.

## Abbreviations

DUS, Doppler ultrasound; rDUS, routine Doppler ultrasound; HAT, hepatic artery thrombosis; PVT, portal vein thrombosis; HVOO, hepatic venous outflow obstruction; iCT, imaging upon indication; CT, computer tomography; LT, liver transplantation; BMI, body mass index; ALD, alcohol related liver disease; SLD, steatotic liver disease; MELD, model for end-stage liver disease; DBD, donation after brain death; DCD, donation after cardiac death.
